# Effect of Temperature of 1.23% Acidulated Phosphate Fluoride Gel on Enamel Microhardness of Permanent Teeth

**DOI:** 10.1155/ijod/8227152

**Published:** 2024-12-05

**Authors:** Shabnam Milani, Mehdi Shahrabi, Nazanin M-Abdi

**Affiliations:** ^1^Department of Pediatric Dentistry, University of British Columbia, Vancouver, Canada; ^2^Department of Pediatric Dentistry, Tehran University of Medical Sciences, Tehran, Iran; ^3^Department of Pediatric Dentistry, Islamic Azad University of Medical Sciences, Tehran, Iran

**Keywords:** acidulated phosphate fluoride, dental caries, dentistry, enamel, hardness, temperature

## Abstract

**Objectives:** This study aimed to assess the effect of temperature of 1.23% acidulated phosphate fluoride (APF) gel on enamel microhardness of permanent teeth.

**Materials and Methods:** This in vitro experimental study was conducted on 36 surgically extracted impacted human third molars. The teeth were mounted in acrylic resin and mesiodistally sectioned into buccal and lingual halves by a high-speed cutting saw. The buccal halves of the teeth were polished with silicone carbide abrasive paper and subjected to pH cycling (8 h of immersion in demineralizing agent and 16 h of immersion in remineralizing agent) for 10 days. Their primary enamel microhardness was subsequently measured by a Vickers microhardness tester. The specimens were then randomly assigned to three groups (*n* = 12) for application of 1.23% APF gel at 4, 25, and 37°C for 4 min. The specimens were then rinsed and incubated for 24 h. Their secondary enamel microhardness was subsequently measured, and the change in microhardness of the specimens was calculated and analyzed by one-way analysis of variance (ANOVA; *α* = 0.05).

**Results:** The mean percentage of change in microhardness was −0.73% ± 16.8%, −5.28% ± 27.32%, and −7.27% ± 32.28% following the application of APF gel at 4, 25, and 37°C, respectively. The difference among the three groups was not significant regarding the percentage of change in microhardness (*p*  > 0.05).

**Conclusion:** Increasing the temperature of 1.23% APF gel from 4 to 25°C and 37°C did not cause a significant change in enamel microhardness of permanent teeth.

## 1. Introduction

Dental caries is the most common infectious disease of the oral cavity which results in loss of hard tooth structure [1]. If not treated early, it can compromise the masticatory function and esthetics. Dental caries is often asymptomatic in the first steps of development. Therefore, patients often seek treatment when the carious lesion has extended to dental pulp and causes pain. Thus, promotion of oral hygiene is imperative to prevent the development or stop the progression of carious lesions [1].

Fluoride is currently the most efficient and most widely used cariostatic agent worldwide [1]. The 4^th^ guideline of the American Dental Association recommends periodic topical fluoride therapy for children and adults at moderate and high risk of caries [1]. The optimal cariostatic property of over-the-counter fluoride products such as toothpastes and mouthwashes and topical fluoride products such as sodium fluoride gel and fluoride varnishes has been well documented [2]. The most important action of fluoride is to increase enamel resistance against acid attacks through the penetration of fluoride ions into the structure of calcium hydroxyapatite and formation of fluorapatite, which increases the resistance and decreases the dissolution of enamel structure [3]. Application of fluoride gel and subsequent fluoride release prevents the development of new carious lesions and stops the progression of existing lesions [4]. Similar to other chemical reactions, this reaction can be influenced by temperature, concentration, and time [5].

Temperature is a material property related to its thermal energy. Increased thermal energy increases the temperature and probably improves the release of fluoride ions from the ionic network, and subsequently enhances their penetration into the structure of calcium hydroxyapatites. Subsequent formation of fluorapatite increases the enamel microhardness as such. Temperature and time are two important factors affecting the intensity of chemical reactions. The positive effect of longer duration of fluoride application on the tooth structure on the uptake of fluoride has been previously reported; however, it does not increase enamel microhardness or its resistance against demineralization [6]. The effect of temperature rise on the release of fluoride ions has also been previously investigated; however, there is a lack of sufficient information regarding the effect of temperature of fluoride product on enamel microhardness.

Hardness is defined as resistance against indentation, deformation, scratching, and surface wear. Increased enamel hardness increases resistance against demineralization [7].

Not much attention has been paid to the temperature of fluoride gels or foams that are commonly used in dental offices. The authors hypothesized that increasing the temperature of fluoride gels may improve their efficacy and increase enamel microhardness. Thus, this study aimed to assess the effect of temperature of 1.23% acidulated phosphate fluoride (APF) gel on enamel microhardness of permanent teeth.

## 2. Materials and Methods

This in vitro experimental study was conducted on 36 human third molars extracted for purposes not related to this study. The patients consented to the use of their extracted teeth for research purposes, and the study protocol was approved by the ethics committee of the university (IR.TUMS.DENTISTRY.REC.1399.027).

### 2.1. Sample Size

The minimum sample size was calculated to be 12 in each group according to a study by Salehzadeh Esfahani, Mazaheri, and Pishevar [2], assuming *α* = 0.05, *β* = 0.2, mean standard deviation of microhardness to be 30 units, and effect size of 56.0 using one-way analysis of variance (ANOVA) power analysis of power analysis sample size (PASS).

### 2.2. Specimen Preparation

The collected teeth were inspected under a microscope to ensure no structural defects. After disinfection in 0.5% chloramine-T solution for 1 week, the teeth were stored in saline at 4°C, and the solution was refreshed weekly until the experiment [2].

The teeth were mounted in acrylic resin and mesiodistally sectioned into buccal and lingual halves by a high-speed cutting machine. The buccal halves were selected for the experiment. For this purpose, they were polished with 1000−2000-grit silicon carbide abrasive papers and stored in distilled water until use [2]. Only the enamel part of the samples was used for further experimentation.

### 2.3. Induction of Artificial Carious Lesions

Tooth blocks underwent pH cycling. For this purpose, they were first immersed in a demineralizing solution composed of 50 mM acetic acid, 100 mM NaCl, 2.2 mM NaH_2_PO_4_, and 12 mM CaCl_2_ with a pH of 4.5 (adjusted by NaOH) for 8 h. Next, they were immersed in a remineralizing solution composed of 3.0 mM NaH_2_PO_4_, 1.0 mM CaCl_2_, and 100 mM NaCl with a pH of 7 for 16 h. This process was repeated for 10 days [2].

### 2.4. Primary Microhardness Testing

The primary enamel microhardness of the specimens was measured after pH cycling by a Vickers microhardness tester (V-Test II, Baresiss, Germany) with 200 g load, which was used to make three indentations on the enamel surface of the prepared samples. The loaded diamond indenter was allowed to penetrate and rest on the enamel surface for 15 s, and the Vickers hardness number was determined and recorded [8].

### 2.5. Application of Fluoride Gel

The specimens were randomly assigned to three groups (*n* = 12) for application of 1.23% APF gel (Avant Dental Supply, USA) at 4, 25, and 37°C. For this purpose, APF gel was applied on the entire enamel surface by a microbrush for 4 min [6]. In group 1, APF gel refrigerated at 4°C was used. In group 2, APF gel kept at room temperature (25°C) was applied, and in group 3, APF gel placed in an incubator at 37°C was used. After 4 min of exposure, the specimens were rinsed and incubated at 37°C and 100% humidity for 24 h [2, 5].

### 2.6. Secondary Microhardness Testing

The specimens underwent a microhardness test again as explained earlier, and subjected to application of 200 g load for 15 s [2].

### 2.7. Statistical Analysis

The data were analyzed using Statistical Package for the Social Sciences (SPSS) version 25 (SPSS Inc., IL, USA). The measures of central dispersion for the primary and secondary microhardness values were reported for all three groups. The Shapiro–Wilk test was used to test the normality and homogeneity of the data. One-way ANOVA was applied to compare the change in microhardness of the three groups at 0.05 level of significance.

## 3. Results

Tables 1 and 2 show the measures of central dispersion for the primary and secondary microhardness values of the three groups, respectively. According to one-way ANOVA, no significant difference was found among the three groups in their primary (*p*=0.95) or secondary (*p*=0.32) microhardness. Since no significant difference was found in general comparisons, no pairwise comparisons were performed.

Table 3 presents the measures of central dispersion for the change in microhardness of the three groups. No significant difference was found among the three groups regarding the change in their microhardness (*p*=0.46).  Table 4 shows the measures of central dispersion for the percentage of change in microhardness of the three groups. No significant difference was detected in this regard among the three groups either (*p*=0.74).

## 4. Discussion

This study assessed the effect of temperature of 1.23% APF gel on enamel microhardness of permanent teeth. The results showed no significant difference among the three groups in the percentage of change in microhardness; although a very slight increase (0.73%) in microhardness was noted in 4°C group, and a small reduction (−5.28% and −7.27%, respectively) was found in 25 and 37°C groups.

When hydroxyapatite reacts with APF, two chemical reactions occur simultaneously: degradation of hydroxyapatite network due to the acidity of the gel and incorporation of fluoride in the composition of fluorapatite [9]. Increasing the temperature from room temperature to 35°C doubles the uptake of fluoride [10]. This increase may occur due to the penetration of fluoride ions into the enamel and enhanced dissolution of enamel due to low pH [11]. Thus, the authors believe that increasing the temperature of APF gel probably increases its molecular activity and acidity of the gel and enables better penetration of fluoride ions into the hydroxyapatite network.

Unlike the present results, Okuno, Nezu, and Tanaka [12] evaluated the effects of topical application of warm fluoride on the human enamel by applying APF gel and 2% and 0.2% NaF at 25, 37, 50, and 60°C for 5 min. They reported that the amount of dissolved fluoride in KOH at 60°C was 1.7 times, 2.6 times, and 2.7 times the rate at 25°C for 0.2% NaF, 2% NaF, and APF gel, respectively. Also, the amount of undissolved fluoride at 60°C was 4.9 times, 7.5 times, and 2.8 times its value at 25°C for 0.2% NaF, 2% NaF, and APF gel, respectively. They recommended warming of topical fluoride solution to increase its efficacy, which was not in line with the present findings probably due to methodological differences between the two studies. Baglar, Nalcaci, and Tastekin [13] evaluated the effect of temperature on fluoride uptake by the enamel using 0.05% NaF mouthwash. They found that the magnitude and speed of fluoride uptake increased by temperature rise. They used a fluoride ion-specific electrode for their measurements and evaluated at 25, 37, and 45°C temperatures, which may explain the difference between their results and the present findings.

In the oral environment, host-related factors such as mineral composition of the tooth and presence of pellicle or dental plaque can affect the rate of demineralization. Saliva-related factors include the saliva flow, composition, and its buffering capacity to protect the teeth [14]. Increasing the remineralizing capacity of the saliva is clinically important. Also, due to the presence of saliva, degree of tooth demineralization in the oral environment is lower than that in vitro.

The present results revealed a reduction in enamel microhardness by an increase in APF gel temperature, although it was not significant. The authors believe that temperature rise increases the acidic effect of APF gel on the enamel and further decreases the enamel microhardness. China et al. [15] evaluated the effects of neutral fluoride and APF gel on microhardness and surface roughness of bleached enamel and reported that application of APF gel along with 35% hydrogen peroxide decreased the enamel microhardness, while application of neutral fluoride along with 35% hydrogen peroxide increased the enamel microhardness. Their results were in line with the present findings.

In the oral environment, the concentration of fluoride in the saliva and dental plaque increases following the application of APF gel, and over time, the released fluoride penetrates into the porosities caused by the acidity of the APF gel. Under such circumstances, application of APF gel increases the uptake of fluoride by the enamel, and improves enamel microhardness as such. Thus, no increase in enamel microhardness following the application of APF gel at different temperatures in the present study may be attributed to the in vitro design of the study.

In the clinical setting, the temperature of fluoride products should be precisely controlled since the temperature cannot exceed 35°C [10]. This factor should be taken into account since further temperature rise may cause pulpal damage [10]. Zach and Cohen [16] showed that surface temperature rise in teeth of Rhesus monkeys by 5.5 and 11.1°C caused irreversible pulpal damage in 15% and 60% of the teeth, respectively. However, Baldissara, Catapano, and Scotti [17] indicated that internal pulpal temperature rise by 8.9 to 14.7°C in humans caused no pathological change in dental pulp. Okuno, Nezu, and Tanaka [12] reported 3 and 8°C pulpal temperature rise following application of 50 and 60°C solutions, respectively. Considering 5.5°C temperature rise as the maximum thermal threshold, increasing the temperature of fluoride solution to 60°C may cause pulpal damage. The safety of using fluoride gels at higher temperatures in the clinical setting is a matter of question and needs further investigations.

In a certain range, higher fluoride uptake has greater clinical benefits for the teeth. Thus, APF solutions with higher fluoride concentration and greater efficacy may be preferred for use. Mellberg and Loertscher [10] reported that at 35°C temperature and a pH of 3, application of 10% fluoride solution increased the fluoride uptake by 119%. However, a slight increase in concentration of fluoride in topical solutions would not be sufficient, and the magnitude of this increase requires further investigations.

The pH of APF gel is in the range of 3–3.5. Maximum fluoride uptake may occur at a pH of 2.5 [10]. Slight differences have been reported in fluoride uptake at pH values of 3 and 4.5 [10]; further increase in pH does not necessarily decrease fluoride uptake. It appears that the main role of low pH in enhancement of fluoride deposition is through the enhancement of hydroxyapatite dissolution, resulting in greater enamel dissolution at a pH range of 3–0 [10]. Considering the significance of the superficial layer in caries progression, assessment of its changes is highly important. The microhardness test is a suitable reproducible method for assessment of surface properties of materials with optimal, nonhomogenous microstructure susceptible to cracks such as enamel [18–20]. The Vickers hardness test used in the present study is suitable for assessment of surface changes at depths <19 µm [21]. The microhardness values obtained in the present study prior to the application of APF gel were in the range of 245–255 kgf/mm^2^, which were within the standard range for normal enamel [22].

This study had some limitations. It had an in vitro design, and complete simulation of the clinical oral environment was not possible. Thus, the results cannot be completely generalized to the clinical setting. In the oral environment, due to the presence of saliva and dental plaque, it is possible that increasing the temperature of APF gel enhances the availability of fluoride and improves the microhardness of tooth structure after a couple of days. However, in the present in vitro study, increasing the temperature probably increased the acidity of the APF gel; however, due to the inability of fluoride reuptake as the result of absence of saliva and dental plaque, the microhardness only slightly decreased [23].

Future studies are required on the effect of temperature rise on the efficacy of other forms of fluoride products such as gels, varnishes, and mouthwashes with different pH values. Moreover, other recently introduced remineralizing agents such as biomimetic hydroxyapatite [24], casein phosphopeptide–amorphous calcium phosphate [25], and calcium sodium phosphosilicate [26] showed promising results. Therefore, the use of these compounds alone or in combination with fluorides should be evaluated in future research. The possible side effects of high temperature of fluoride products should be investigated as well. Furthermore, artificial saliva can be used in future studies to better simulate the clinical setting. Clinical studies are also required to obtain more reliable results. Additionally, the correlation of microhardness data with polarized light microscopy findings should be evaluated in further studies.

## 5. Conclusion

Increasing the temperature of 1.23% APF gel from 4 to 25°C and 37°C did not cause a significant change in enamel microhardness of permanent teeth.

## Figures and Tables

**Table 1:** Measures of central dispersion for the primary microhardness (kgf/mm^2^) of the three groups.

Group (°C)	Mean	Std. deviation	Std. error	95% CI		*p* value
				Lower bound	Upper bound	
4	250.19	40.88	11.8	224.22	276.17	0.95
25	247.83	45.07	13.01	219.19	276.47	
37	254.42	65.42	18.89	212.85	295.98	

Abbreviations: CI, confidence interval; Std., standard.

**Table 2:** Measures of central dispersion for the secondary microhardness (kgf/mm^2^) of the three groups.

Group (°C)	Mean	Std. deviation	Std. error	95% CI		*p* value
				Lower bound	Upper bound	
4	248.97	42.06	12.14	222.25	275.69	0.32
25	225.17	32.35	9.34	204.61	245.72	
37	223.64	57.44	16.58	187.15	260.13	

Abbreviations: CI, confidence interval; Std., standard.

**Table 3:** Measures of central dispersion for the change in microhardness (kgf/mm^2^) of the three groups.

Group (°C)	Mean	Std. deviation	Std. error	95% CI		*p* value
				Lower bound	Upper bound	
4	−1.22	39.13	11.29	−26.08	23.64	0.46
25	−22.67	65.49	18.9	−64.27	18.94	
37	−30.78	68.67	19.82	−74.41	12.86	

Abbreviations: CI, confidence interval; Std., standard.

**Table 4:** Measures of central dispersion for the percentage of change in microhardness of the three groups.

Group (°C)	Mean	Std. deviation	Std. error	95% CI		*p* value
				Lower bound	Upper bound	
4	−0.73	16.8	4.85	−9.94	11.41	0.74
25	−5.28	27.32	7.89 K	−22.64	12.08	
37	−7.27	32.28	9.32	−27.78	13.24	

Abbreviations: CI, confidence interval; Std., standard.

## Data Availability

The data used to support the findings of this study were supplied by the corresponding author under license and data will be available on request. Requests for access to these data should be made to the corresponding author.
